# Regulation of the expression of nine antimicrobial peptide genes by *Tm*IMD confers resistance against Gram-negative bacteria

**DOI:** 10.1038/s41598-019-46222-8

**Published:** 2019-07-12

**Authors:** Yong Hun Jo, Bharat Bhusan Patnaik, Jihun Hwang, Ki Beom Park, Hye Jin Ko, Chang Eun Kim, Young Min Bae, Woo Jin Jung, Yong Seok Lee, Yeon Soo Han

**Affiliations:** 1 Division of Plant Biotechnology, Institute of Environmentally-Friendly Agriculture (IEFA), College of Agriculture and Life Sciences, Chonnam National University, Gwangju, 61186 Republic of Korea; 2 School of Biotech Sciences, Trident Academy of Creative Technology (TACT), Chandrasekharpur, Bhubaneswar, Odisha 751024 India; 3 Department of Agricultural Chemistry, Institute of Environmentally-Friendly Agriculture (IEFA), College of Agriculture and Life Sciences, Chonnam National University, Gwangju, 61186 Korea; 4 Department of Life Science and Biotechnology, College of Natural Sciences, Soonchunhyang University, Asan city, 336-745 Republic of Korea

**Keywords:** Inhibitory RNA techniques, Innate immunity

## Abstract

Immune deficiency (IMD) is a death domain-containing protein that is essential for the IMD/NF-κB humoral and epithelial immune responses to Gram-negative bacteria and viruses in insects. In the immune signaling cascade, IMD is recruited together with FADD and the caspase DREDD after the mobilization of PGRP receptors. Activated IMD regulates the expression of effector antimicrobial peptides (AMP) that protect against invading microorganisms. To date, most studies of the IMD pathway, and the *IMD* gene in particular, have been restricted to *Drosophila;* few similar studies have been conducted in other model insects. Herein, we cloned and functionally characterized an *IMD* homolog from the mealworm beetle *Tenebrio molitor* (*Tm*IMD) and studied its role in host survival in the context of pathogenic infections. Phylogenetic analysis revealed the conserved caspase cleavage site and inhibitor of apoptosis (IAP)-binding motif (IBM). *TmIMD* expression was high in the hemocytes and Malpighian tubules of *Tenebrio* late-instar larvae and adults. At 3 and 6 hours’ post-infection with *Escherichia coli*, *Staphylococcus aureus*, or *Candida albicans*, *TmIMD* expression significantly increased compared with mock-infected controls. Knockdown of the *TmIMD* transcript by RNAi significantly reduced host resistance to the Gram-negative bacterium *E*. *coli* and fungus *C*. *albicans* in a survival assay. Strikingly, the expression of nine *T. molitor* AMPs (Tm*Tenecin1*, Tm*Tenecin2*, Tm*Tenecin4*, Tm*Defensin2*, Tm*Coleoptericin1*, Tm*Coleoptericin2*, Tm*Attacin1a*, Tm*Attacin1b*, and Tm*Attacin2*) showed significant downregulation in *TmIMD* knockdown larvae challenged with *E*. *coli*. These results suggest that *Tm*IMD is required to confer humoral immunity against the Gram-negative bacteria, *E*. *coli* by inducing the expression of critical transcripts that encode AMPs.

## Introduction

Insects represent the largest group within the arthropod phylum. Over 50% of the described eukaryotes are insects, which inhabit variety of habitats and interact with diverse surrounding environments^1^. The innate immune system of insects is specialized and is considered as one of the major attributable factors for the robustness and consistency of species across ecosystems. The innate immune system of insects recognizes and removes microbial threats through cellular and systemic immune responses. Cellular responses include phagocytosis mediated by plasmatocytes (these cells can be compared to white blood cells in humans) and lamellocytes^2^. Systemic responses (including local responses in the epithelia of the skin and intestine) are mediated by antimicrobial peptides (AMPs) produced in the fat body (equivalent to the vertebrate liver) of insects^3^. The AMPs are transcriptionally regulated by the Toll and immune deficiency (IMD) signaling pathways, in addition to the Janus Kinase/signal transducer and activator of transcription (JAK/STAT) and c-Jun N-terminal kinase (JNK) pathways that are conspicuous and evolutionarily conserved across insect taxa^4^. The core Toll and IMD signaling pathways act synergistically in insects to activate innate immune responses towards microorganisms via the production of lysozymes, AMPs, and other effector proteins. These synergistic interactions demonstrate cross-regulation of signaling processes that can lead to a broad-spectrum host response. AMPs cloned from many insect models including *Drosophila*, have revealed the conserved nuclear factor kappaB (NF-κB) binding sites in their promoter elements, suggesting the evolutionary conservation of the AMP production mechanisms and effector actions^5^. Although both the Toll and IMD pathways are important in controlling the expression of AMPs, the IMD pathway in particular is essential as it regulates the expression of most AMPs in *Drosophila*^6^. By contrast the Toll pathway is involved in embryonic axis formation and controls a limited set of AMPs^7,8^.

The IMD signaling pathway is activated after the recognition of Gram-negative bacteria or viruses^9,10^. In *Drosophila*, the IMD signaling pathway is activated through the recruitment of the death domain-containing intracellular protein and Fas-associated protein with death domain (FADD) as well as the caspase death-related ced-3/Nedd2-like protein (DREDD). This occurs as a consequence of bacterial peptidoglycan (PGN)-mediated multimerization and clustering of the transmembrane receptor peptidoglycan recognition protein-LC (PGRP-LC)^11,12^. DREDD cleavesIMD protein at the caspase cleavage site, creating a novel binding site for inhibitor of apoptosis-2 (IAP-2). The E2-ubiquitin-conjugating enzyme complex (UEV1a, Ubc13, and Ubc5) associates with the IAP-2 binding site and activates the TGF-beta-activated kinase 1 binding protein 2/Mitogen-activated protein kinase kinase kinase 7 (Tab2/Tak1) complex^11,13^. Subsequently, this complex drives phosphorylation of the IκB kinase (IKK) complex (ird5 and key) to activate Relish. After translocating to the nucleus, Relish activates the transcription of AMP genes that are essential for humoral and epithelial immune responses^14,15^.

In the context of IMD signaling, the IMD protein acts as an adapter molecule (similar to mammalian receptor interacting proteins) in the antibacterial defense system of insects. This has been established in the *Drosophila* model in which the overexpression of *IMD* led to the constitutive expression of AMP genes^16^. Studies of the IMD pathway, especially of *IMD*, have been largely restricted to the *Drosophila* model system, as limited studies have been conducted in other insect models. Although many homologs of the IMD pathway including the *IMD* gene, have been discovered in other insects through high-throughput RNA sequencing (RNA-seq)^17,18^, the mechanisms that regulate AMP expression needs to be further explored. In one recent study, the requirement of *Bombyx mori* *IMD* (*BmIMD*) in the induction of *Drosomycin*, *DiptericinA*, and *DiptericinB* was studied via overexpression in *Drosophila* S2 cells^19^. In the red flour beetle *Tribolium castaneum*, the IMD pathway was proposed to confer resistance against the Gram-negative and Gram-positive pathogens *Enterobacter cloacae* and *Bacillus subtilis*, respectively, through the robust induction of AMP genes. In *T*. *castaneum* pupae, the RNAi mediated knockdown of *IMD* transcripts led to downregulation of AMPs, such as *Attacin1* and *Coleoptericin1*, at 24 h post-infection with *Escherichia coli*^20^.

In the *Tenebrio* model system, limited information is available about genes in the IMD pathway and the regulation of AMP gene expression. This is in contrast to the elegant studies that have been conducted on the Toll signaling pathway in model insects. For example, the extracellular signaling cascades involving proteases and the Toll ligand Spätzle have been elucidated^21,22^. Additionally, intracellular signaling cascades emanating from the Toll -Spätzle complex and the antimicrobial responses have been partially explored^23,24^. In this study, we report for the first time, cloning of the full-length cDNA of the *T*. *molitor IMD* gene (*TmIMD*) and the analysis of its sequence features and phylogenetic relationships at the amino acid level. The temporal and spatial expression profiles of *TmIMD* transcripts and its response to bacterial (*E*. *coli* and *S. aureus*) and fungal (*Candida albicans*) challenges were characterized at multiple time-points. We used RNAi to understand the requirement of *TmIMD* transcripts for survival of *T. molitor* larvae and AMP gene expression. A set of nine AMP genes was found to be downregulated in the *E*. *coli* infection model after the silencing of *TmIMD* transcripts. Our study advances an understanding of the IMD regulatory pathway in the innate immune response of *T*. *molitor* through the regulation of expression of AMP genes.

## Results

### Gene organization, cDNA sequence analysis, and phylogenetics

**Figure 1:**
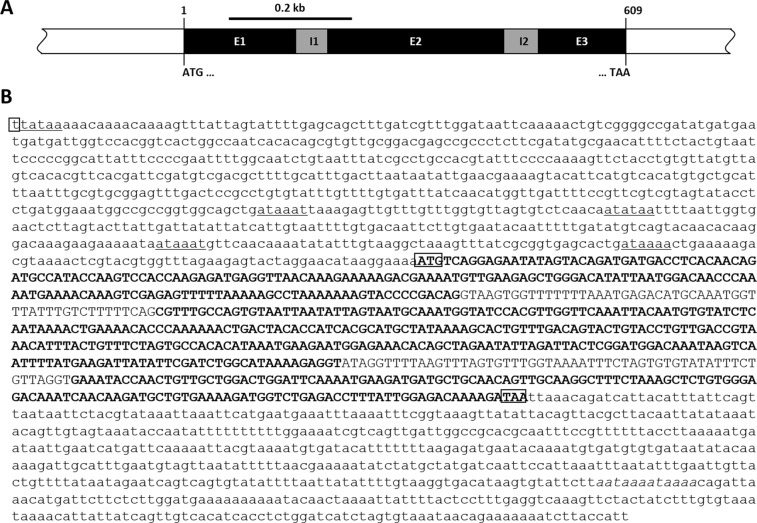
Genomic organization of the *T*. *molitor IMD* (*TmIMD*) gene. (**A**) Schematic representation of the *TmIMD* gene. The genomic structure (open bar) shows three exons (black) interspersed with two introns (grey). The translation start (ATG) and stop (TAA) codons are marked demarcating the ORF region. (**B**) The sequence of the *TmIMD* gene with the ORF region (capitalized). Exonic regions are in bold. Transcription start, translation start codon, and translation stop codons are boxed. The poly(A) sequence is indicated in italics. In the mRNA, the poly(A) begins after the last nucleotide in exon 3.

**Figure 2:**
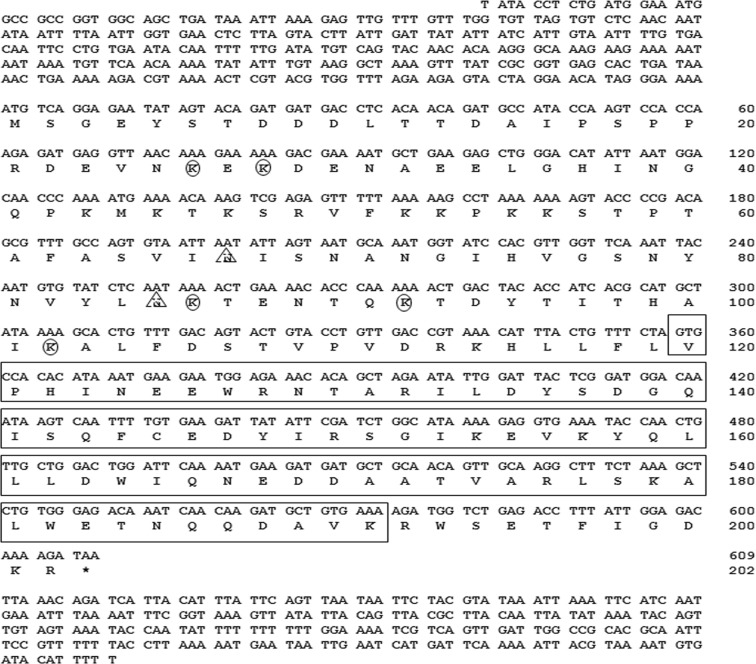
Nucleotide and deduced amino acid sequences of *TmIMD*. The nucleotides are numbered from the first base of the ORF region represented by the start codon. The death domain extending from position 120 to 191 is shown in an open box. The potential N-glycosylation and polyubiquitination sites are indicated with triangles (N67 and N85) and circles (K26, K28, K86 and K92), respectively.

We used a standalone local blast server (downloaded from ftp://ftp.ncbi.nlm.nih.gov/blast/executables/blast+/) and the *T*. *molitor* RNAseq and expressed sequence tag libraries (unpublished) to retrieve the *TmIMD* sequence. Local tblastn analysis was conducted using the *Tribolium castaneum* *IMD* (*TcIMD*) sequence as the query (NCBI Reference Sequence XM_008201183.2). We retrieved one full-length *TmIMD* ORF sequence. This sequence was used as a query against the *T*. *molitor* DNAseq database (unpublished) as the subject in a blastn analysis to identify the *TmIMD* genomic sequence. Analyzing the gene structure of *TmIMD*, we identified three exons and two introns (Fig. 1A). A detailed study on the *TmIMD* gene organization is shown in Fig. 1B. The exons are 181 bp (exon 1), 286 bp (exon 2), and 142 bp (exon 3), while the introns 1 and 2 are 52 and 56 bp, respectively. The transcription start site (t) was identified at 751 bp upstream of the translation start codon (ATG) and the poly(A) sequence was found at 448 nucleotides downstream of the translation stop codon (TAA). The ORF sequence contains 609 nucleotides and encodes a 202 amino acid residues (Fig. 2). A death domain (72 amino acids), similar to that of the mammalian receptor-interacting protein (RIP), was predicted at the C-terminus of the protein. This domain is evolutionarily conserved in many multicellular organisms and is critical for the recruitment of the downstream effector molecules involved in apoptotic and immune signaling pathways^25^. N-glycosylation sites were predicted at N67 and N85, while four polyubiquitination sites were predicted at K26, K28, K86, and K92. No O-glycosylation sites were predicted for *Tm*IMD, in contrast to six O-glycosylation sites in the IMD homolog of *B*. *mori*^19^. The *TmIMD* nucleotide and translated amino acid sequence was deposited in GenBank (through BankIt submission) under the accession number MK121950.

A blastp search of the *Tm*IMD amino acid sequence performed against the NCBI non-redundant database retrieved several other proteins showing homology to IMD proteins in insects and other organisms. Multiple alignment analysis of *Tm*IMD with other insect homologs showed that the amino acids D and A at the interface of caspase cleavage and the IAP-binding motif (IBM) were conserved (Fig. S1). These form the caspase cleavage site critical for IMD cleavage by DREDD in a FADD-dependent manner as well as the downstream regulation of the transcription factor Relish and induction of the expression of AMPs^26^. Furthermore, most of lepidopteran insect sequences showed a conserved ‘LKSDA’ motif, whilst in the coleopterans it was ‘LTTDA’ and in dipterans ‘LETDA’ (and was ‘LEKDA’ in *Drosophila* isoforms).

**Figure 3:**
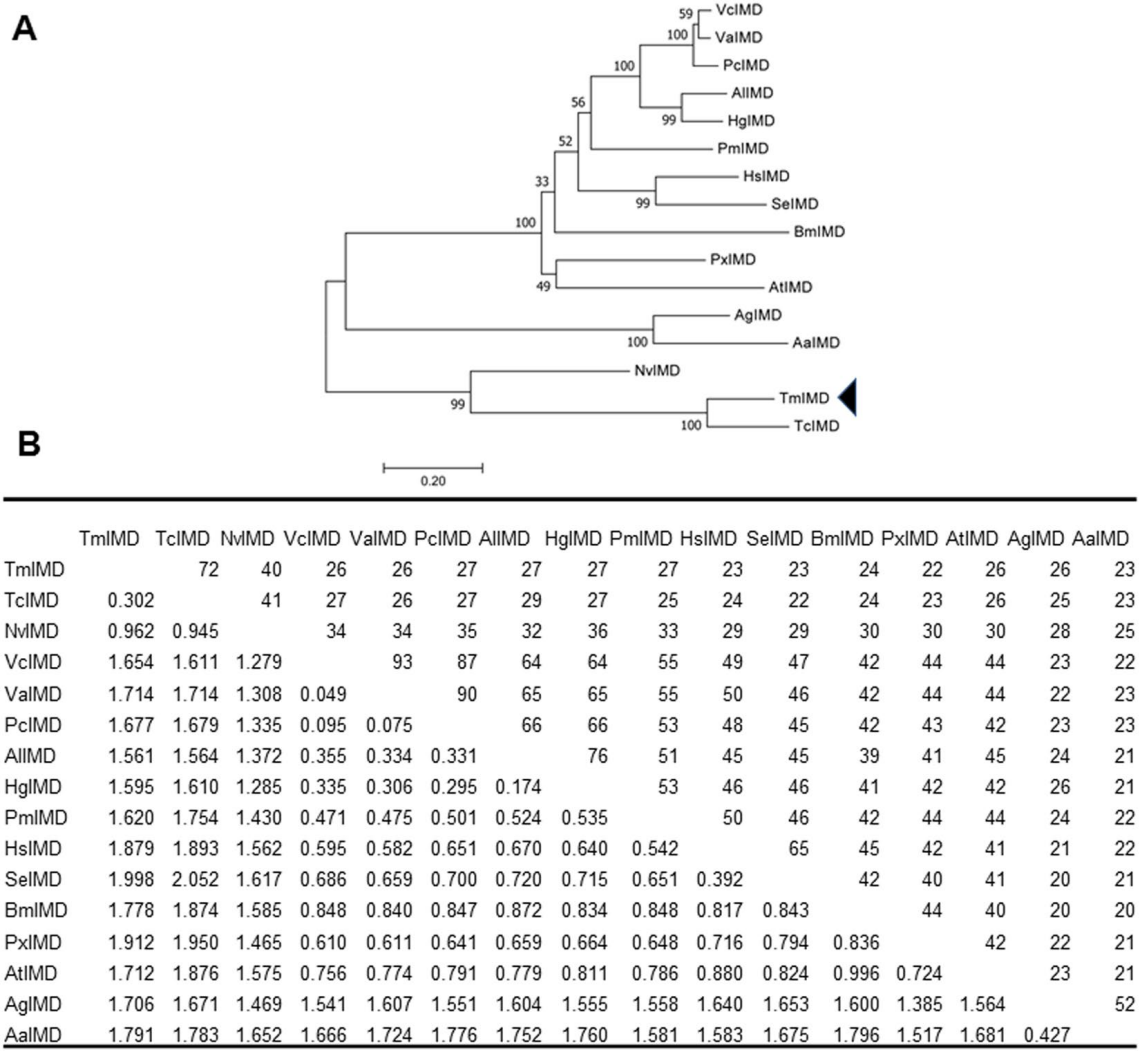
Phylogenetic tree and percentage identity/distance matrix of *Tm*IMD. (**A**) The unrooted Neighbor- Joining phylogenetic tree of *Bm*IMD and orthologous proteins from insects based on the JTT matrix model. The percentage of replicate trees in which the associated taxa clustered together in the bootstrap test (1,000 replicates) is shown next to the branches. An arrow head indicates *Tm*IMD. (**B**) Estimates of evolutionary divergence between sequences of *Tm*IMD and orthologous proteins from insects are illustrated by percentage identity (upper block) and distance matrix (lower block). The abbreviations and GenBank accession numbers (in parenthesis) are: *Tc*IMD, *T. castaneum* IMD (XP_008199405); *Dm*IMD-A, *D. melanogaster* IMD, isoform A (NP_573394.1); *Dm*IMD-B, *D. melanogaster* IMD, isoform B (NP_001286572.1); *Hg*IMD, *Hebomoia glaucippe* IMD (AFK75936.1); *Ag*IMD, *A. gambiae* (XP_001688608); *Pc*IMD, *Polygonia c-album* IMD (AFK75940.1); *Vc*IMD, *Vanessa cardui* IMD (AFK75942.1); *Am*IMD, *A. mellifera* IMD (NP_001157189.1); *Va*IMD, *Vanessa atalanta* IMD (AFK75941.1); *Aa*IMD, *A. aegypti* AAEL010083-PA (EAT37980.1); *Al*IMD, *Appias lyncida* IMD (AFK75935.1); *Sg*IMD, *Schistocerca gregaria* IMD-like protein (AFK75938.1); *Hs*IMD, *Heliothis subflexa* IMD (AFK75939.1); *Px*IMD, *Plutella xylostella* IMD (AFK75937.1); and *Nv*IMD, *Nasonia vitripennis* IMD (NP_001135910.1).

A phylogenetic tree was constructed using IMD amino acid sequences from 16 representative insect species, including *Drosophila* IMD isoform A and B (*Dm*IMD-A & *Dm*IMD-B) (Fig. 3A). The results revealed the distribution of the IMD sequences into three major groups comprising of Lepidoptera, Coleoptera/Hymenoptera, and Diptera. *Tm*IMD was firstly grouped with its close relative *Tc*IMD protein, and then the members of this coleopteran group was clustered into the branch of hymenopterans comprising of *Nasonia vitripennis* IMD (*Nv*IMD) and *Apis mellifera* IMD (*Am*IMD) proteins. Among the Dipterans, the *Dm*IMD-A and *Dm*IMD-B sequences were identical, while *Anopheles gambiae* IMD (*Ag*IMD) and *Aedes aegypti* IMD (*Aa*IMD) were found to be related. The evolutionary divergence matrix also revealed less than 25% identity of *Tm*IMD with IMD proteins from Lepidoptera and Diptera orders (Fig. 3B). Considering the multiple alignment and divergence matrix of IMD sequences from the orthologs, it is evident that there is a selection pressure to maintain the caspase/IBM motifs in *Tm*IMD, whereas the other sequences do not show the same signature of selection.

### Temporal and spatial expression of *TmIMD* transcripts

**Figure 4:**
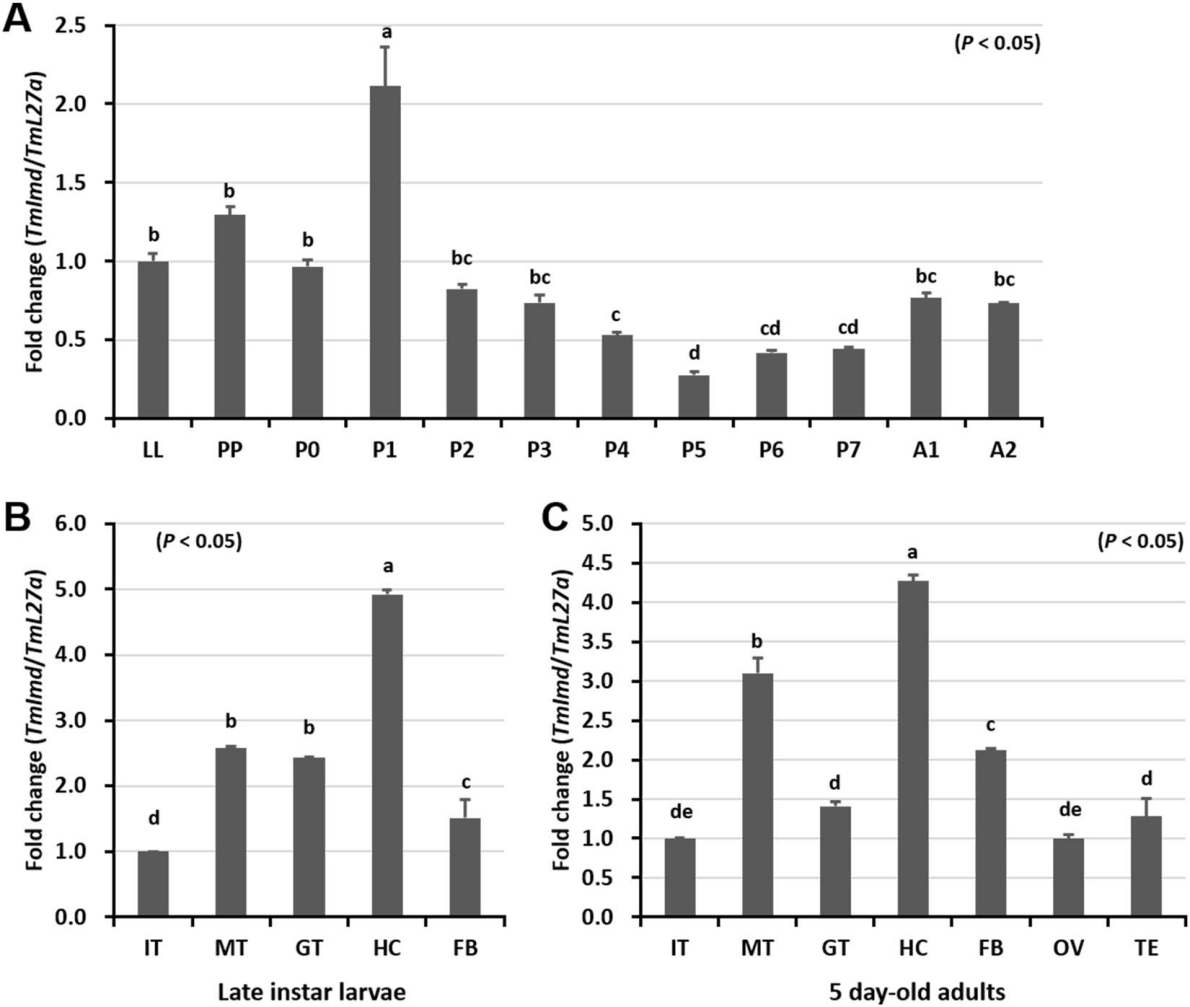
Developmental and tissue distribution of *TmIMD*. (**A**) The mRNA transcript levels of *TmIMD* in different developmental stages of the insect were analyzed using qPCR. LL: late-instar larva; PP: pre-pupa; P0–P7: zero to seven-day old pupa; A1-A2: one to two-day old adult. (**B**) The mRNA transcript levels of *TmIMD* in larval and adult tissues of the insect were analyzed by qPCR. IT: integument; MT: Malpighian tube; GT: gut; HC: hemocytes; FB: fat body; OV: ovary; TE: testis. *T. molitor Ribosomal protein L27a* (*TmL27a*) was used as an internal control. Vertical bars represent standard errors (n = 3). One-way ANOVA and Tukey’s multiple range tests at 95% confidence level (*P* < 0.05) were performed and used to determine the level of significance of differences.

We analyzed the expression patterns of *TmIMD* mRNA transcripts in different days of larval, pupal, and adult development using qRT-PCR. The expression of *TmIMD* mRNA was observed in all the developmental stages of the insect, but was more pronounced in the late larval, prepupal, and very early pupal stages (P0 and P1) (Fig. 4A). Expression data in Fig. 4 refer to the late larval stage (set to 1.0). The expression levels of *TmIMD* mRNA transcripts peaked at stage P1 (close to the larval-pupal transition). In the mid- and late-pupal stages, the mRNA transcript levels of *TmIMD* were found to be lower compared to the other stages, with the lowest levels at stage P5. In the A1 and A2 stages, the expression of *TmIMD* mRNA was high, but non-significant compared to other stages, except for the P1 and P5 stages.

To gain further insights into the function of *TmIMD* in different tissues of *T*. *molitor* larvae and adults, we examined the expression patterns of *TmIMD* mRNA by qRT-PCR analysis. Here, *TmIMD* expression referred to the expression in the integument (set to 1.0). We found that *TmIMD* was expressed in all of the tested tissues of *T*. *molitor* larva and adult. The expression levels of *TmIMD* mRNA were the highest in the hemocytes compared to the expression levels in all other larval tissues analyzed (Fig. 4B). In other tissues, such as the fat body and gut that are involved in systemic and local immune responses, respectively, the expression levels of *TmIMD* mRNA was also found to be higher. In the adult tissues of *T*. *molitor*, the expression levels of *TmIMD* mRNA were found to be the highest in the hemocytes, followed by the expression in Malpighian tubules and fat body, respectively (Fig. 4C).

### Microbial challenge-induced expression of *TmIMD* transcripts

**Figure 5:**
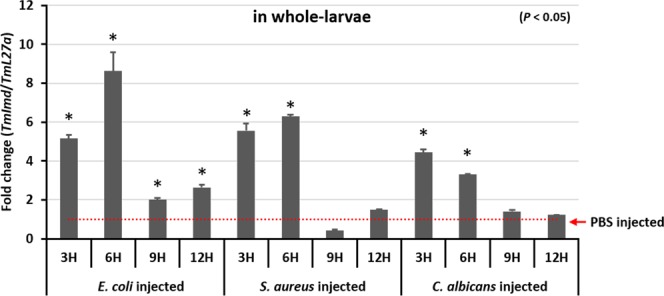
The mRNA transcript levels of *TmIMD* in whole larvae (10–12^th^ instar) after microbial challenge. After challenge injections of *E*. *coli*, *S*. *aureus*, or *C*. *albicans*, the mRNA transcript levels of *TmIMD* were analyzed by qPCR. *TmL27a* was used as an internal control. Results of triplicate experiments are shown with standard errors (n = 3). Asterisks indicate significant differences at *P* < 0.05 between control (PBS) and microbial challenge groups for each time point.

The changes in expression of *TmIMD* mRNA were analyzed in response to immune challenges with the Gram-negative bacteria *E*. *coli*, Gram-positive bacteria *S*. *aureus*, and fungus *C*. *albicans* (Fig. 5). *TmIMD* mRNA increased more than 8-fold 6 h after the injection of *E*. *coli*. In *S*. *aureus-*injected *T*. *molitor* larvae, *TmIMD* mRNA was increased 6-fold at 6 h. These results were found to be significant at 95% confidence levels. Even after the injection of the fungus, there was a significant expression of *TmIMD* transcripts. In our time-course, *TmIMD* transcripts decreased by 12 h after microbial challenge. These early induction patterns in the microbial challenge studies suggests a putative role of *TmIMD* in systemic immune signaling.

### *TmIMD in vivo* knockdown assay

**Figure 6:**
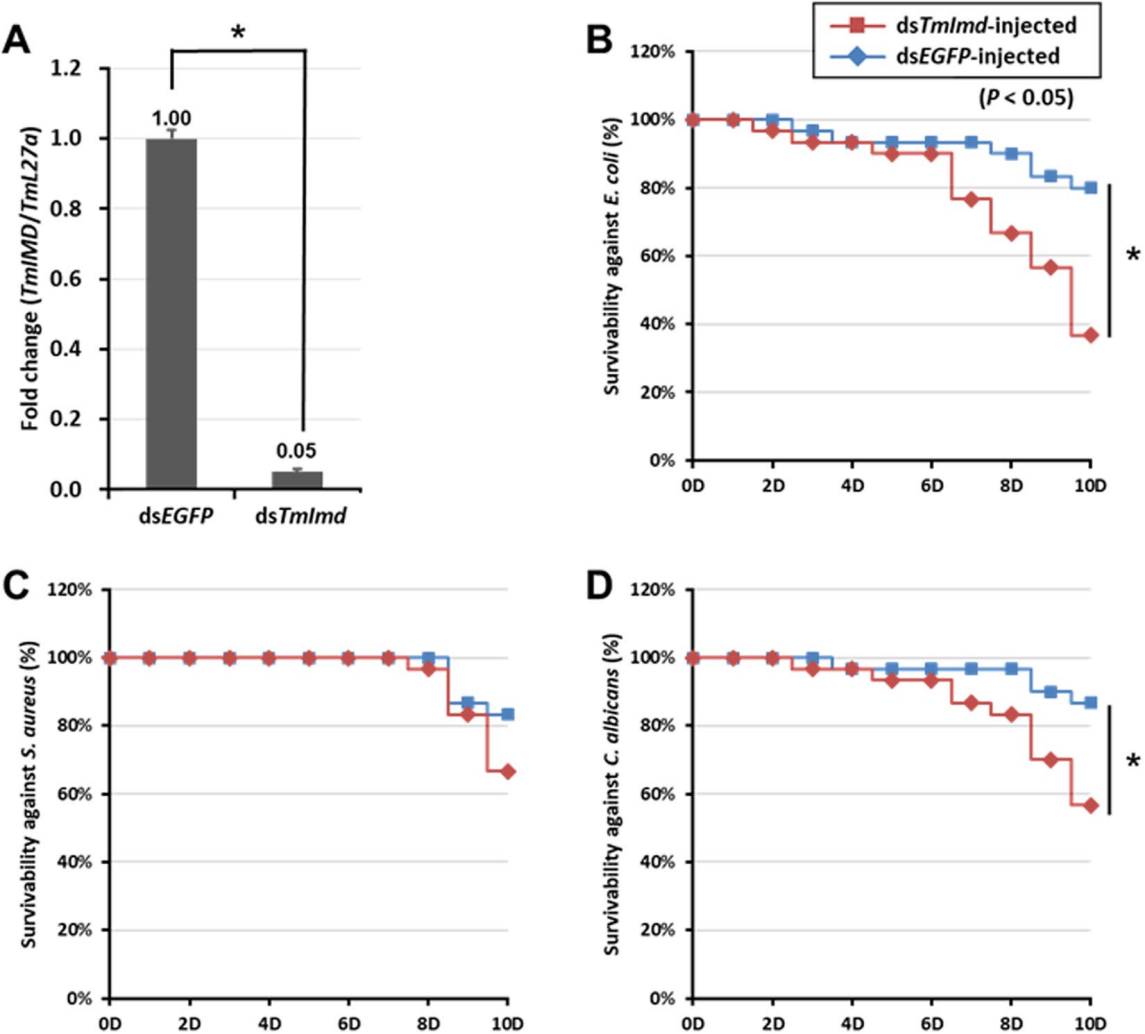
RNAi knockdown of *TmIMD* enhanced the susceptibility of *T*. *molitor* larvae to *E*. *coli* and *C*. *albicans* infection. (**A**) *T*. *molitor* larvae (10–12^th^ instar) were injected with ds*TmIMD* (2 µg per insect) and transcript silencing was measured by qPCR. The *EGFP* dsRNA-treated larvae served as a negative control. Data represent means ± standard error of three independent biological replicates. *E*. *coli* (**B**), *S*. *aureus* (**C**), and *C*. *albicans* (**D**) were inoculated at 2-days post RNAi, and larval survival was recorded daily for 10 days in each treatment group. The experiment was repeated three times with similar results. Statistical analysis of survival analysis was carried out based on Kaplan-Meier plots (log-rank chi-square test; *p < 0.05).

To determine whether *TmIMD* is involved in conferring immune response against bacterial and fungal challenge, we generated double-stranded RNA (dsRNA) against *TmIMD* and knocked it down in systemic circulation in the *T*. *molitor* whole-larvae. After confirmation of efficient knockdown (generally 2 days after dsRNA treatment), we investigated the lethality rates of *TmIMD* knockdown larvae following *E*. *coli*, *S*. *aureus*, and *C*. *albicans* challenge using larval mortality assays. We constructed a dsRNA corresponding to a 363 bp portion of the *TmIMD* gene. A dsRNA for the *Enhanced Green Fluorescent Protein* (*EGFP*) sequence (derived from plasmid EGFP-C1) was also synthesized to act as a negative control in the survival analysis. The dsRNA for the *EGFP* sequence corresponded to a 546 bp portion of the ORF sequence. We confirmed efficient knockdown (95%) of *TmIMD* mRNA by qRT-PCR (Fig. 6A). We find that the knockdown efficiency of *TmIMD* mRNA was stable at least for a duration of 10 days from day 2 after ds*TmIMD* injection. Next, we challenged *TmIMD* knockdown larvae with *E*. *coli*, *S*. *aureus*, or *C*. *albicans*, and subsequently analyzed the survival of larvae for the duration of 10 days. The survival analysis was not continued beyond 10 days as *TmIMD* knockdown was not active. The survival of the infected ds*EGFP*-injected *T*. *molitor* was considered for comparison. Of note, there was a 40% reduction in survival compared to controls in *IMD* knockdown beetles subjected to *E*. *coli* infection (Fig. 6B). A non-significant 15–20% reduction in the survival of larvae was observed in the case of *S*. *aureus* infection (Fig. 6B), and a significant 40% reduction was observed in the case of *C*. *albicans* infection (Fig. 6C). Considering the survival analysis, *TmIMD* was confirmed as a positive regulator because its knockdown resulted in increased susceptibility against the Gram-negative bacteria *E*. *coli* and the fungus *C*. *albicans*. However, *TmIMD* is not directly involved in immune response against *S*. *aureus*.

### AMP gene expression analysis

**Figure 7:**
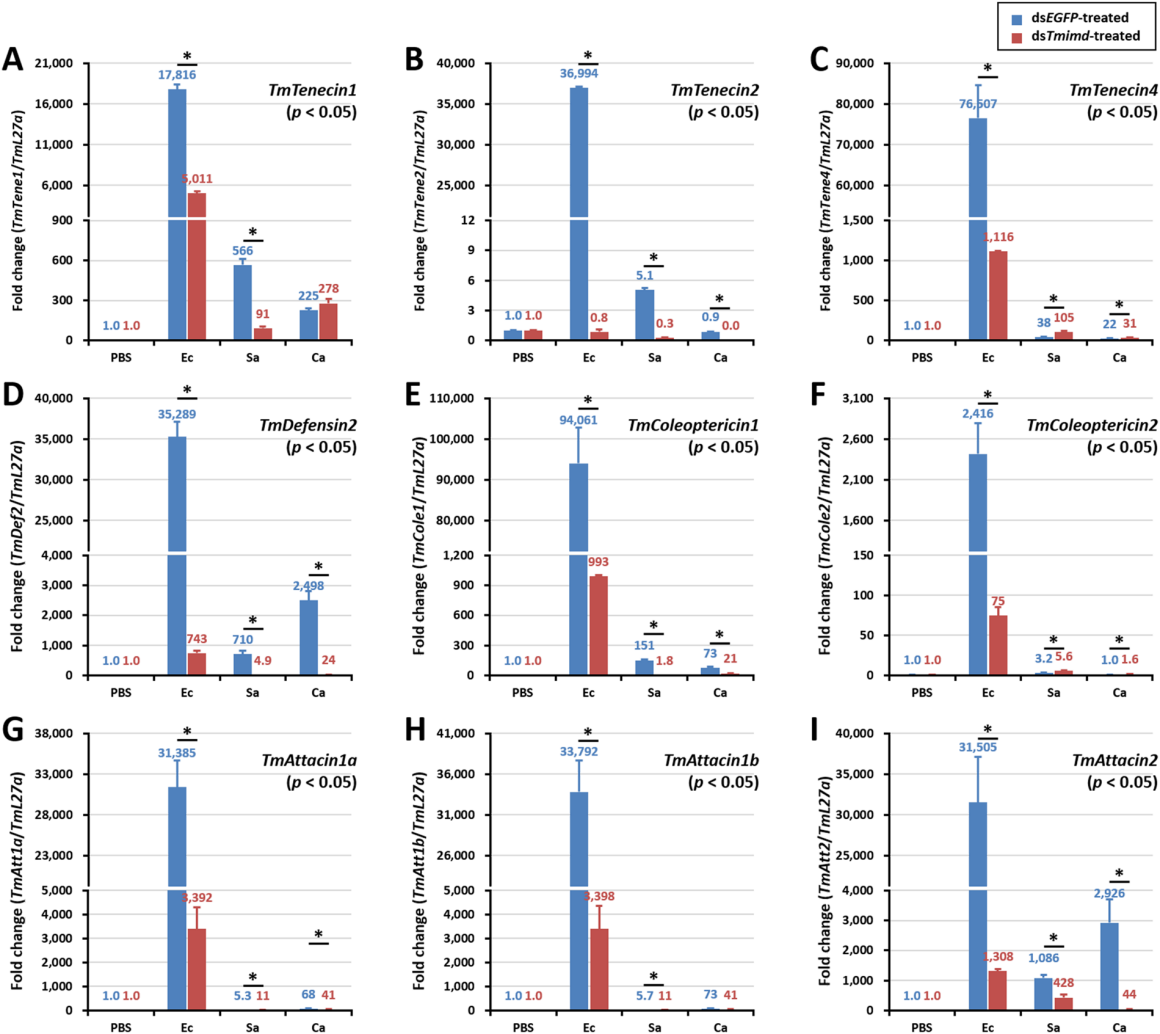
AMP expression levels in *TmIMD-*knockdown *T*. *molitor* larvae upon microorganism challenge. *E*. *coli* (Ec), *S*. *aureus* (Sa), or *C*. *albicans* (Ca) were injected into ds*TmIMD*-treated *T*. *molitor* larvae. The mRNA transcript levels of *TmTenecin1* (**A**), *TmTenecin2* (**B**) *TmTenecin4* (**C**), *TmDefensin2* (**D**), *TmColeoptericin1* (**E**), *TmColeoptericin2* (**F**), *TmAttacin1a* (**G**), *TmAttacin1b* (**H**), and *TmAttacin2* (**I**) were analyzed by qPCR. *EGFP* dsRNA was used as a silencing control and *TmL27a* was used as an internal control. *TmIMD* knockdown showed impaired expression of AMPs upon *E*. *coli* infection in *T*. *molitor* larvae. Results show an average of three independent biological replicates with standard errors. All data were analyzed for significance using Student’s *t*-test (*p < 0.05).

To investigate the involvement of *Tm*IMD in humoral immunity, we measured the transcript levels of 14 *Tenebrio* AMPs after microbial challenge in *in vivo TmIMD* silenced whole-larvae by qRT-PCR. The level of expression of AMPs were compared between *TmIMD* knockdown and *EGFP* control groups. The *T*. *molitor* AMPs such as *Tm*Tenecin, *Tm*Defensin, *Tm*Cecropin, *Tm*Thaumatin-like protein, *Tm*Coleoptericin, and *Tm*Attacin were considered for the studies. After confirmation of *in vivo* knockdown (2 days after dsRNA treatment), we injected larvae with *E*. *coli*, *S*. *aureus*, *C*. *albicans*, or phosphate buffered saline (PBS). After one day of microbial infection, AMP gene expression was analyzed. We found that from among the 14 AMPs analyzed, the expression levels of 9 AMPs were significantly upregulated (i.e., greater than 2000-fold) in ds*EGFP*-injected larvae. Except for *TmTenecin3*, *TmDefensin1*, *TmCecropin2*, *TmThaumatin-likeprotein1*, and *TmThaumatin-like protein2*, other AMPs showed very high transcriptional upregulation. *TmColeoptericin1* (Fig. 7E) and *TmTenecin4* (Fig. 7C) showed the maximum expression followed by *TmTenecin2* (Fig. 7B), *TmDefensin2* (Fig. 7D), *TmAttacin1b* (Fig. 7H), *TmAttacin2* (Fig. 7I), *TmAttacin1a* (Fig. 7G), *TmColeoptericin2* (Fig. 7F), and *TmTenecin1* (Fig. 7A). In the *dsTmIMD-* injected larvae, expression levels of all of these 9 AMPs were significantly reduced after *E*. *coli* infection, explaining the larval susceptibility to the Gram-negative bacterial infection. Hence, it is confirmed that *TmIMD* is required for AMP induction and humoral immunity functions after Gram-negative bacterial infections. Further, AMPs such as *TmTenecin3* (Fig. S2A), *TmThaumatin-like protein1* (Fig. S2D), *TmDefensin1* (Fig. S2B), *TmThaumatin-like protein2* Fig. S2E), and *TmCecropin2* (Fig. S2C) were not detected or were slightly induced in larvae treated with *dsEGFP*. In contrast, knock-down of *IMD* increased *TmDefensin1* expression by more than 8,500-fold in *E*. *coli-*challenged conditions. In the *C*. *albicans* infection group, only *TmDefensin2* and *TmAttacin2* showed a significant reduction in expression under *TmIMD* knockdown conditions.

## Discussion

In this study, we identified a full-length ORF of the *TmIMD* gene in the coleopteran beetle *T*. *molitor* using bioinformatics analysis. The full-length ORF of *TmIMD* was then queried against the *T*. *molitor* genome database to retrieve the *TmIMD* genomic sequence. All IMD homologs in different insect species are 240–280 amino acids long. The *Tm*IMD amino acid sequence was comparatively shorter to IMD-like proteins from most other Lepidopteran and Dipteran species. The coleopteran IMD homologs lack sequences that correspond to the non-conserved regions in IMD proteins but showed a completely conserved structure at the caspase cleavage site and the IBM motif. In *Tm*IMD, the putative cleavage site occurs at _10_LTTD/A_15_. In the *Drosophila* IMD protein, the caspase cleavage site (_27_LEKD/A_31_) was found to be critical for DREDD- mediated IMD cleavage in an FADD- dependent manner, which then drives further downstream signaling events^12^. Moreover, the cleavage is necessary to expose a neo N-terminus with a highly conserved IBM motif that binds the Death-associated inhibitor of apoptosis 2 (E3 ligase DIAP2) via its baculovirus IAP repeat 2/3 domains^27^. For the IMD pathway in *Drosophila*, the DIAP2-IMD interaction is rapidly ubiquitinated at the K63 position, and the polyubiquitin chains serve to recruit the TAB2/TAK1 complex^28^. Unlike the K63 polyubiquitination site in *Drosophila*, a potential polyubiquitination site at K92 occurs in *Tm*IMD and *BmIMD*. Hence, it can be speculated that *Tm*DREDD could cleave *Tm*IMD in a *Tm*FADD-dependent manner leading to the recruitment of the *Tm*TAB2/*Tm*TAK1 complex. This could subsequently activate *Tm*Relish for downstream induction of AMPs. Similar studies have been attempted in the red flour beetle *T*. *castaneum* to understand the host defense mechanism against bacteria^20,29^, taking the advantage of the *T*. *castaneum* genome annotation (BioProjects: PRJNA15718, PRJNA12540)^30^. Taken together, we have identified the *IMD* gene from *T*. *molitor* and characterized the protein in detail. Because limited studies have been conducted on the functional characterization of IMD homologs and activation of the IMD-Relish-AMPs cascade in insects, our study should help to advance our understanding of innate immunity among insects.

**Figure 8:**
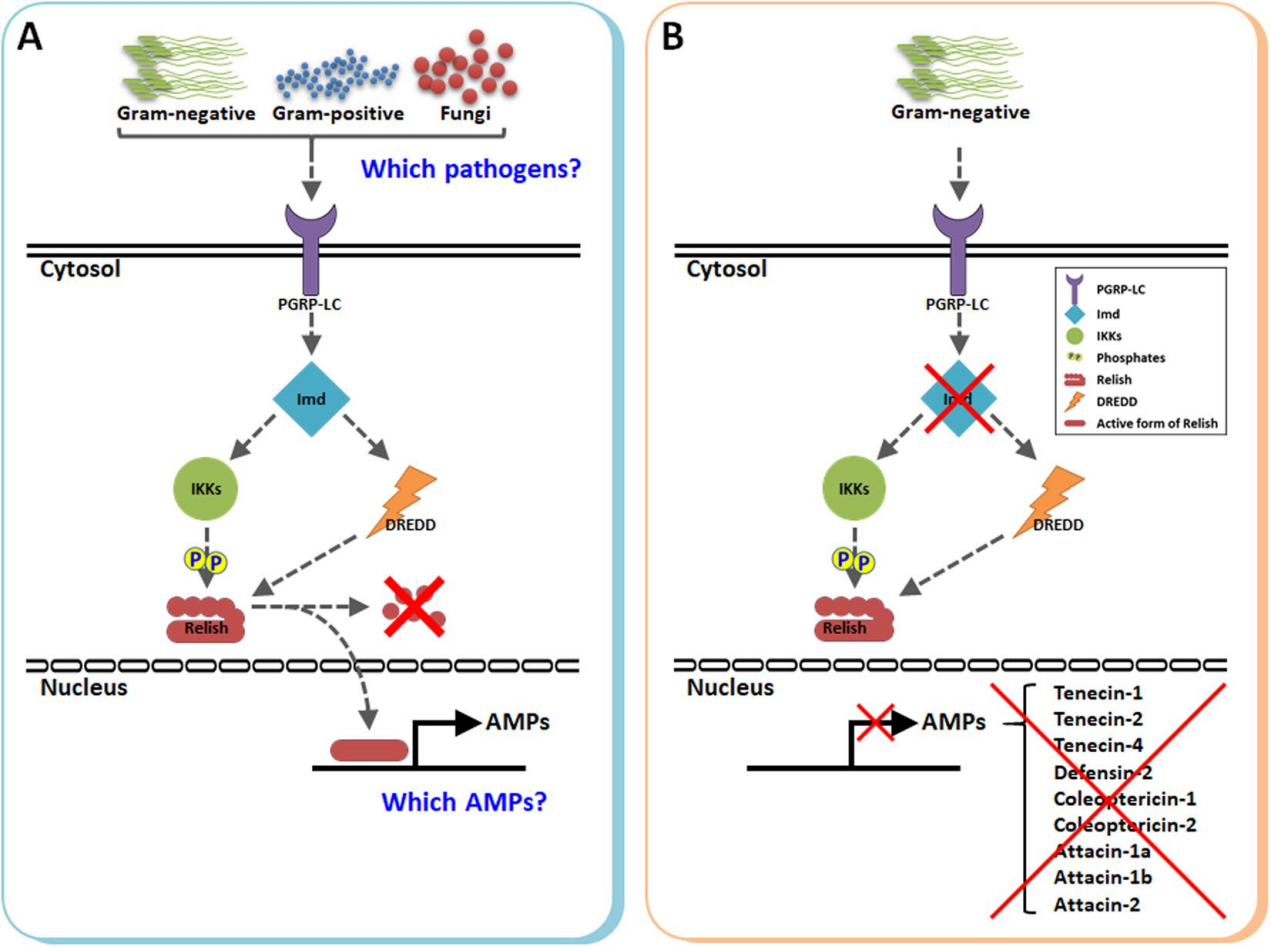
A putative model for *Tm*IMD-mediated regulation of AMPs in response to the Gram-negative bacteria *E*. *coli*. (**A**) In *Tenebrio*, PGRP-LC senses bacterial and/or fungal pathogens and processes the signal via the cleavage of the cytoplasmic adapter protein IMD. Subsequently, IMD drives the phosphorylation of the NF-κB homolog in the IMD pathway, Relish. Activated Relish translocates to the nucleus and controls the transcriptional activation of specific AMP genes. However, the nexus between the pathogen types that regulate the expression of specific sets of AMP genes remains obscure. (**B**) We propose that the knockdown of *IMD* transcripts prevents the activation and subsequent translocation of Relish to the nucleus, thereby downregulating the expression of nine AMP genes in the *Tenebrio* model system.

This study showed that the mRNA expression levels of *TmIMD* are upregulated at 3–6 h after *E*. *coli*, *S*. *aureus*, or *C*. *albicans* challenge compared to a healthy wounding control. The expression of *BmIMD* is activated at the midgut level during oral infection with *E*. *coli* or *S*. *aureus*^19^. Irrespective of the mode of infection by microorganisms, IMD is implicated as a regulator of innate immune signaling in insect models. To understand how this occurs in *T*. *molitor*, we designed *TmIMD-*knockdown *T*. *molitor* larvae and studied larval mortality after infection with microorganisms. The RNAi technique is quite popular for characterizing cell signaling pathways triggered by a specific class of pathogen. In the *Drosophila* model, the components of the IMD pathway were analyzed after the synthesis of nearly 7,000 dsRNAs^31^. Furthermore, the technique has been employed to understand the molecular interactions and effector functions of extracellular and intracellular Toll signaling pathways in *T*. *molitor*^23,32^. In the present study, we found that *T*. *molitor* larvae are susceptible to *E*. *coli* infection with a mortality percentage of >60% in the *TmIMD*- knockdown cohort. A 40% mortality of larvae were also observed in the context of fungal infection. But in case of *S*. *aureus* infection, the mortality % in the *TmIMD* knockdown larvae was non-significant compared with the ds*EGFP* treated group. This suggests that *Tm*IMD contributes to the innate immune response against the Gram-negative bacteria *E*. *coli* and the fungus *C*. *albicans*. Further in the intestinal infection model of *D*. *melanogaster*, IMD pathway is indispensable to the innate immunity process^33^. In a previous study, we reported the involvement of *Tm*MyD88 in the positive regulation of host immune responses against *S*. *aureus* using RNAi^23^. MyD88 is an adapter protein in the Toll signaling pathway that mediates immune responses against most Gram-positive bacteria, including *S*. *aureus*^34^. Hence, we speculate that the induction of *Tm*IMD after *S*. *aureus* challenge is not necessarily related with the immune responses and overall survival of *T*. *molitor* larvae. In systemically infected insect models, Gram-positive bacterial infections are mostly regulated by the Toll signaling pathway, but in the gut-specific immune responses in *Drosophila*, the IMD pathway is implicated for the clearance of Gram-positive bacteria *S*. *aureus*^35^. The *IMD* knockdown pupae of *T*. *castaneum* failed to mount an appropriate immune response against *E*. *coli* or *B. subtilis* infection^36^. Furthermore, in the *Drosophila* model, IMD pathway components were found to be proactively involved in promoting host resistance against the bacterium *S. marcescens*^37^. Understanding the preferential regulation of AMP genes under the influence of the Toll or IMD pathway is important in the context of infection models because these pathways are suggested to act synergistically and show cross-regulation^20,38^. A study has attributed the presence of GGGGATTYYY as the kB consensus motif for IMD-responsive genes and GGGAAADYCC for Toll-responsive genes, explaining the differential regulation of AMPs^39^. In one of our earlier studies on the *T*. *molitor* Toll signaling pathway, we confirmed the role of the Cactin protein in regulating the expression of seven AMP genes in response to *E*. *coli* and *S aureus*^32^. This study for the first time explains the *E*. *coli-*induced AMP gene expression under a *TmIMD* knockdown condition in *T*. *molitor* larvae. The significantly strong and robust expression of nine *Tenebrio* AMPs (*TmColeoptericin1*, *TmTenecin4*, *TmTenecin2*, *TmTenecin1*, *TmDefensin2*, *TmAttacin1b*, *TmAttacin2*, *TmAttacin1a*, and *TmColeoptericin2)* in an *E*. *coli-* challenged condition indicates that these AMPs are required to elicit a host defense response against infection. Further, in the *TmIMD-*knockdown insects, these AMPs showed a significant reduction in expression, suggesting that in *T*. *molitor*, *TmIMD* is required to regulate the expression of these AMPs and mount a host-protective response against the Gram-negative bacteria *E*. *coli* (Fig. 8). Nevertheless, it will be interesting to determine whether all upregulated AMPs show activity against such infective agents. The moderate expression of 2 AMPs (*TmDefensin2 and TmAttacin2*) was also observed in a *C*. *albicans-* challenged condition, indicating the involvement of these AMPs in the immune response of *T*. *molitor* against the fungi. Moreover, a downregulation in the expression of these AMPs after *TmIMD* knockdown suggests a possible requirement of this gene in regulating the AMPs and defense against fungal infection. AMPs, such as *TmTenecin3*, *TmDefensin1*, *TmCecropin2*, *and TmThaumatin-like protein1/2-*, showed low to very low expression in *T*. *molitor* larvae under infective conditions. This is surprising because *TmTenecin3* contributes to host defense against fungal infections^40^. In *T*. *castaneum*, *IMD* knockdown reduced the expression levels of *T*. *castaneum Attacin1*, *Defensin2*, *Defensin3*, and *Coleoptericin1* after *E*. *coli* challenge. However, that study was conducted in the context of understanding the preferential induction of AMP genes in response to microbial challenges by using RNAi to target either MyD88 (a Toll pathway adaptor protein) or *IMD*^20^. Irrespective of the dominant role of the IMD pathway in contributing to defense against *E*. *coli*, we also speculate the promiscuous role of the Toll pathway in directing the expression of AMPs via Dif- Relish heterodimer formation because the DAP-type peptidoglycan in Gram-negative bacteria is known to activate the Toll ligand Spätzle and induce defensin-like AMPs in *Tenebrio* larvae^22^.

## Materials and Methods

### *T. molitor* and microbial strains

Larvae of the mealworm beetle, *T*. *molitor*, were reared in our laboratory with an artificial diet (4.4 g NeoVita, 0.5 g chloramphenicol, 0.4 g L-ascorbic acid, 0.5 g sorbic acid, 0.5 ml propionic acid, 2.2 g yeast extract, 2.2 g bean powder, 7.6 g agar, 4.4 g wheat powder, and 73 g wheat bran in 200 ml deionized water; autoclaved at 121 °C for 15 min) at 26 ± 1 °C, and 60% ± 5% relative humidity under dark conditions. In all experiments, only healthy 10–12^th^ instar larvae were taken. Gram-positive bacteria, *Staphylococcus aureus*, strain RN4220 (also referred to as NRS144) were purchased from BEI Resources, NIAID, NIH, USA. *Escherichia coli* K12; Strain SMG 123 (PTA-7555) were procured from the American Type Culture Collection (ATCC). *C*. *albicans* was cultured in Sabouraud Dextrose broth, while overnight cultures of *E*. *coli* and *S*. *aureus* were cultured in Luria- Bertani (LB) broth at 37 °C. For the injection experiments, microorganisms were harvested and washed in phosphate-buffered saline (PBS; pH 7.0) by centrifugation at 3,500 *g* for 10 min. The washed samples were resuspended in PBS and concentrations at OD_600_ were measured. The final concentrations were adjusted to 10^6^ cells/µl for *E*. *coli* and *S*. *aureus* and 5 × 10^4^ cells/µl for *C*. *albicans*. The concentrations of the microorganisms have been validated in *T*. *molitor* larvae for immune challenge experiments as reported in previous studies^32^.

### Identification of *TmIMD* and *in silico* analysis

The full-length *TmIMD* gene was identified by taking the *T*. *castaneum IMD* (*TcIMD*) amino acid sequence as the query (XP_008199405) and the *T*. *molitor* nucleotide database derived from the RNAseq/EST (unpublished) as the subject using local tblastn analysis. To further understand the genomic organization of *TmIMD*, we conducted local-blastn analysis using the obtained nucleotide sequence of *TmIMD* as the query and the *T*. *molitor* DNAseq database (unpublished) as the subject. Subsequently, the *Tm*IMD ORF sequence was mapped in the genomic sequences using the eukaryotic gene-finding program FGENESH^41^. The conserved domains/motifs were predicted using InterProScan 5.0^42^ and blastp programs. N-glycosylation and potential ubiquitination sites were predicted using the NetPhos 3.1 Server^43^ and UbPred^44^, respectively. The multiple alignment, percentage identity, and distance analysis of *Tm*IMD amino acid and orthologous sequences from different orders were conducted using the ClustalX2 program^45^ and visualized using Genedoc software (http://iubio.bio.indiana.edu/soft/molbio/ibmpc/genedoc-readme.html). The phylogenetic tree was constructed based on the maximum likelihood tree method using the MEGA 7.0 program^46^.

### Gene expression analysis and immune challenge studies

Reverse transcription quantitative PCR (RT-qPCR) was performed using the Exicycler^TM^ real-time Quantitative Thermal Block (Bioneer co., Daejon, South Korea) to analyze developmental and tissue distributions of *TmIMD* at the mRNA transcript level. In brief, total RNA was isolated (using 6 M Guanidine hydrochloride, 20 mM each of 0.5 M EDTA and 1 M 2-(N-morpholino) ethanesulfonic acid, and 40 µM phenol red) from different developmental stages, such as late larvae (10^th^–12^th^ instar larvae; lengths of approximately 3 cm), pre-pupae, pupae (1–7 days old), and adults (1–2 days old). For tissue-specific expression profiles, total RNA was isolated from the larval tissues, such as integument, gut, fat body, Malpighian tubules, and hemocytes, and adult tissues, such as integument, gut, fat body, Malpighian tubules, hemocytes, ovary, and testes after disrupting the tissues with Precellys Evolution Homogenizer (Bertin Instruments, Montigny-le-Bretonneux, France) Notably, hemocytes were collected as pellets after centrifugation of the hemolymph at 5,000 × *g* for 4 min. For gene expression analysis, 15 insects were used (five insects were pooled together as one group). From each of the groups, tissue samples were collected to make three samples from each tissue.

For challenge experiments, healthy *T*. *molitor* larvae were microinjected intra-abdominally with 10^6^ cells/µl of *E*. *coli* and 5 × 10^4^ cells/µl of *S*. *aureus*, or *C*. *albicans*, respectively. PBS was used as a wounding control. Larvae (n = 3) were collected at 3, 6, 12, and 24 h postinjection (p.i) and total RNA was extracted at each time point using FavorPrep^TM^ Tri-RNA reagent (Favorgen biotech corp., Ping-Tung, Taiwan). For cDNA synthesis, 2 µg total RNA was used with AccuPower RT Pre-Mix (Bioneer) and Oligo (dT)_12–18_ primer. The real-time PCR assay was performed at an initial denaturation of 95°C for 20 s, followed by 45 cycles of 95°C for 5 s, and 60°C for 20 s using AccuPower 2X GreenStar qPCR Master Mix (Bioneer). *Ribosomal protein L27a* (*RpL27a*) was used as an internal control for normalizing the expression results obtained in the experimental groups. Experiments were performed in triplicate and are presented as mean ± standard error of three biological replications.

### *TmIMD* gene silencing

For *TmIMD* RNAi, a 363 bp PCR product was amplified by Ex Taq^TM^ Polymerase (Takara, Japan) using specific primers containing the T7 promoter sequences at the 5′-ends. As a negative control, a 546 bp PCR product of the *EGFP* gene (derived from plasmid EGFP-C1) was used as a template for *EGFP* RNA synthesis. The primers for RNAi (Table 1) were designed using the SnapDragon-Long dsRNA design software available at DRSC/TRiP functional genomics resources (https://www.flyrnai.org/cgi-bin/RNAi_find_primers.pl) to minimize the off-target effects, if any. PCR was conducted at an initial denaturation of 98°C for 5 min, followed by 30 cycles of denaturation at 98°C for 10 s, annealing at 55°C for 30 s and extension at 72°C for 1 min. The purified PCR product was used to synthesize *TmIMD* double-strand RNA (dsRNA) using an AmpliScribe T7-Flash Transcription Kit (Epicentre Biotechnologies, Madison, WI, USA) following the manufacturer’s instructions. Briefly, the 20 µl reaction mixture was incubated at 42°C for 16 h, followed by 75°C for 5 min, and 25°C for 30 min. The dsRNA was purified using PCI (Phenol: Chloroform: Isoamyl alcohol), precipitated using 5 M ammonium acetate and ethanol, and resuspended in molecular biology grade water. The sample was measured spectrophotometrically at 260 nm and the integrity was determined by agarose gel electrophoresis. A total of 2 µg (in 1 µl) synthesized *TmIMD* dsRNA was injected intra-abdominally into the 10^th^–12^th^ stage larvae of *T*. *molitor*. The *dsEGFP* was injected into the host using a disposable needle mounted onto a Picospritzer III micro-dispense system (Parker). The *EGFP* dsRNA was injected as a negative control in RNAi studies. For each gene, at least 30 last-instar larvae (10^th^–12^th^ instar) were injected. The experiment was conducted in triplicate to confirm the silencing of *TmIMD* transcripts.

**Table 1:** Used primer sequences.

Name	Primer sequences
TmIMD sqPCR-Fw TmIMD sqPCR-Rev	5-AGGCTAAAGTTTATCGCGGTGA-3′ 5′-CGGCCAATCAACTGACGATT-3′
dsTmIMD-Fw dsTmIMD-Rev	5′-TAATACGACTCACTATAGGGTGACAGCGTTTGCCAGTGTAA-3′ 5′-TAATACGACTCACTATAGGGTTGAATCCAGTCCAGCAACAG-3′
dsEGFP-Fw dsEGFP-Rev	5′-TAATACGACTCACTATAGGGTACGTAAACGGCCACAAGTTC-3′ 5′-TAATACGACTCACTATAGGGTTGCTCAGGTAGTGTTGTCG-3′
TmIMD qPCR-Fw TmIMD qPCR-Rev	5′-ACCAAGTCCACCAAGAGATGAG-3′ 5′-TTACACTGGCAAACGCTGTC-3′
TmL27a qPCR-Fw TmL27a qPCR-Rev	5′-TCATCCTGAAGGCAAAGCTCCAGT-3′ 5′-AGGTTGGTTAGGCAGGCACCTTTA-3′
TmTenecin1-Fw TmTenecin1-Rev	5′-CAGCTGAAGAAATCGAACAAGG-3′ 5′-CAGACCCTCTTTCCGTTACAGT-3′
TmTenecin2-Fw TmTenecin2-Rev	5′-CAGCAAAACGGAGGATGGTC-3′ 5′-CGTTGAAATCGTGATCTTGTCC-3′
TmTenecin3-Fw TmTenecin3-Rev	5′-GATTTGCTTGATTCTGGTGGTC-3′ 5′-CTGATGGCCTCCTAAATGTCC-3′
TmTenecin4-Fw TmTenecin4-Rev	5′-GGACATTGAAGATCCAGGAAAG-3′ 5′-CGGTGTTCCTTATGTAGAGCTG-3′
TmDefensin1-Fw TmDefensin1-Rev	5′-AAATCGAACAAGGCCAAACAC-3′ 5′-GCAAATGCAGACCCTCTTTC-3′
TmDefensin2-Fw TmDefensin2-Rev	5′-GGGATGCCTCATGAAGATGTAG-3′ 5′-CCAATGCAAACACATTCGTC-3′
TmColeoptericin1-Fw TmColeoptericin1-Rev	5′-GGACAGAATGGTGGATGGTC-3′ 5′-CTCCAACATTCCAGGTAGGC-3′
TmColeoptericin2-Fw TmColeoptericin2-Rev	5′-GGACGGTTCTGATCTTCTTGAT-3′ 5′-CAGCTGTTTGTTTGTTCTCGTC-3′
TmAttacin1a-Fw TmAttacin1a-Rev	5′-GAAACGAAATGGAAGGTGGA-3′ 5′-TGCTTCGGCAGACAATACAG-3′
TmAttacin1b-Fw TmAttacin1b-Rev	5′-GAGCTGTGAATGCAGGACAA-3′ 5′-CCCTCTGATGAAACCTCCAA-3′
TmAttacin2-Fw TmAttacin2-Rev	5′-AACTGGGATATTCGCACGTC-3′ 5′-CCCTCCGAAATGTCTGTTGT-3′
TmThaumatin-like protein1-Fw TmThaumatin-like protein1-Rev	5′-CTCAAAGGACACGCAGGACT-3′ 5′-ACTTTGAGCTTCTCGGGACA-3′
TmThaumatin-like protein2-Fw TmThaumatin-like protein2-Rev	5′-CCGTCTGGCTAGGAGTTCTG-3′ 5′-ACTCCTCCAGCTCCGTTACA-3′

^※^Underline indicates T7 promotor sequences.

### Survival analysis

As a measure of the mortality under *TmIMD* knockdown conditions, 10^6^ cells/µl of *E*. *coli* and 5 × 10^4^ cells/µl of *S*. *aureus* and *C*. *albicans* were injected to separate sets of larvae. The survival analysis was conducted with 10 insects for each set and results represented an average of three biological replicates. To monitor mortality, larvae that died during the first 3 h of microorganism challenge were discarded. Surviving larvae were incubated at 26 °C and scored daily for a period of 10 days.

### AMP expression analysis

To understand the role of *TmIMD* transcripts in regulating the expression of 14 AMP genes, including *TmTenecin1*, *TmTenecin2*, *TmTenecin3*, *TmTenecin4*, *TmDefensin1*, *TmDefensin2*, *TmColeoptericin1*, *TmColeoptericin-2*, *TmAttacin1a*, *TmAttacin1b*, *TmAttacin2*, *TmCecropin2*, *TmThaumatin-like protein1*, and *TmThaumatin-like protein2*, microorganisms were injected into *TmIMD-*knockdown *T*. *molitor* larvae. A *EGFP* dsRNA was used as a negative control and PBS was used as an injection control. Samples at 12 h post injection of microorganisms were processed for total RNA analysis, cDNA synthesis, and qPCR analysis using AMP primers (Table 1).

### Statistical analysis

For qPCR-based expression analysis of *TmIMD* transcripts, the 2^−ΔΔCt^ method^47^ was used. Data were subjected to one-way analysis of variance (ANOVA) and Tukey’s multiple range tests to evaluate differences between groups (*P* < 0.05). For the survival analysis, a Kaplan-Meier plot (log-rank chi-square test) was generated using Real Statistics using Excel (http://www.real-statistics.com/survival-analysis/kaplan-meier-procedure/real-statistics-kaplan-meier/).

## Conclusions

This study is the first to identify and functionally characterize the IMD homologue from the coleopteran pest *T. molitor*. We found that *Tm*IMD is required for innate immune-mediated host defense of *T*. *molitor* larva against the Gram-negative bacteria *E*. *coli* and the fungus *C*. *albicans*. Furthermore, systemic *E*. *coli* infection in *T. molitor* larvae with *TmIMD* knockdown downregulates the production of a specific set of AMP genes that includes *TmTenecin1*, *TmTenecin2*, *TmTenecin4*, *TmDefensin2*, *TmColeoptericin1*, *TmColeoptericin2*, *TmAttacin1a*, *TmAttain1b*, *and TmAttacin2*. In future studies, it will be interesting to note the functional roles of downstream regulatory molecules in IMD signaling pathway, such as FADD, DREDD, IKKγ, TAB2/TAK1, and Relish, in the context of innate immune-mediated host defense against microorganisms, and to study the conservation of IMD pathways across insect species.

## Supplementary information

Supplementary Figure 1 and 2
