# Supplementary material for: Regulation of the expression of nine antimicrobial peptide genes by TmIMD confers resistance against Gram-negative bacteria
Source: Sci Rep. 2019 Jul 12;9:10138. doi: 10.1038/s41598-019-46222-8 (PMC6626034; doi:10.1038/s41598-019-46222-8)
Supplement: Supplementary file 1 — Supplementary Figure 1 and 2 [file 41598_2019_46222_MOESM1_ESM.docx]

**Regulation of the expression of nine antimicrobial peptide genes by *Tm*IMD confers resistance against Gram-negative bacteria**

Yong Hun Jo^1#^, Bharat Bhusan Patnaik^1,2#^, Jihun Hwang^1^, Ki Beom Park^1^, Hye Jin Ko^1^, Chang Eun Kim^1^, Young Min Bae^1^, Woo Jin Jung^3^, Yong Seok Lee^4^, and Yeon Soo Han^1*^

^1^Division of Plant Biotechnology, Institute of Environmentally-Friendly Agriculture (IEFA), College of Agriculture and Life Sciences, Chonnam National University, Gwangju 61186, Republic of Korea.

^2^School of Biotech Sciences, Trident Academy of Creative Technology (TACT), Chandrasekharpur, Bhubaneswar, Odisha, 751024, India

^3^Department of Agricultural Chemistry, Institute of Environmentally-Friendly Agriculture (IEFA), College of Agriculture and Life Sciences, Chonnam National University, Gwangju 61186, Korea

^4^Department of Life Science and Biotechnology, College of Natural Sciences, Soonchunhyang University, Asan city 336-745, Republic of Korea

* Corresponding author

Yeon Soo Han,

Address: Division of Plant Biotechnology, Institute of Environmentally-Friendly Agriculture (IEFA), College of Agriculture and Life Sciences, Chonnam National University, Gwangju 61186, Republic of Korea

E-mail: hanys@jnu.ac.kr

Tel.: +82-62-530-2072; Fax: +82-62-530-2069

# These authors contributed equally to this work


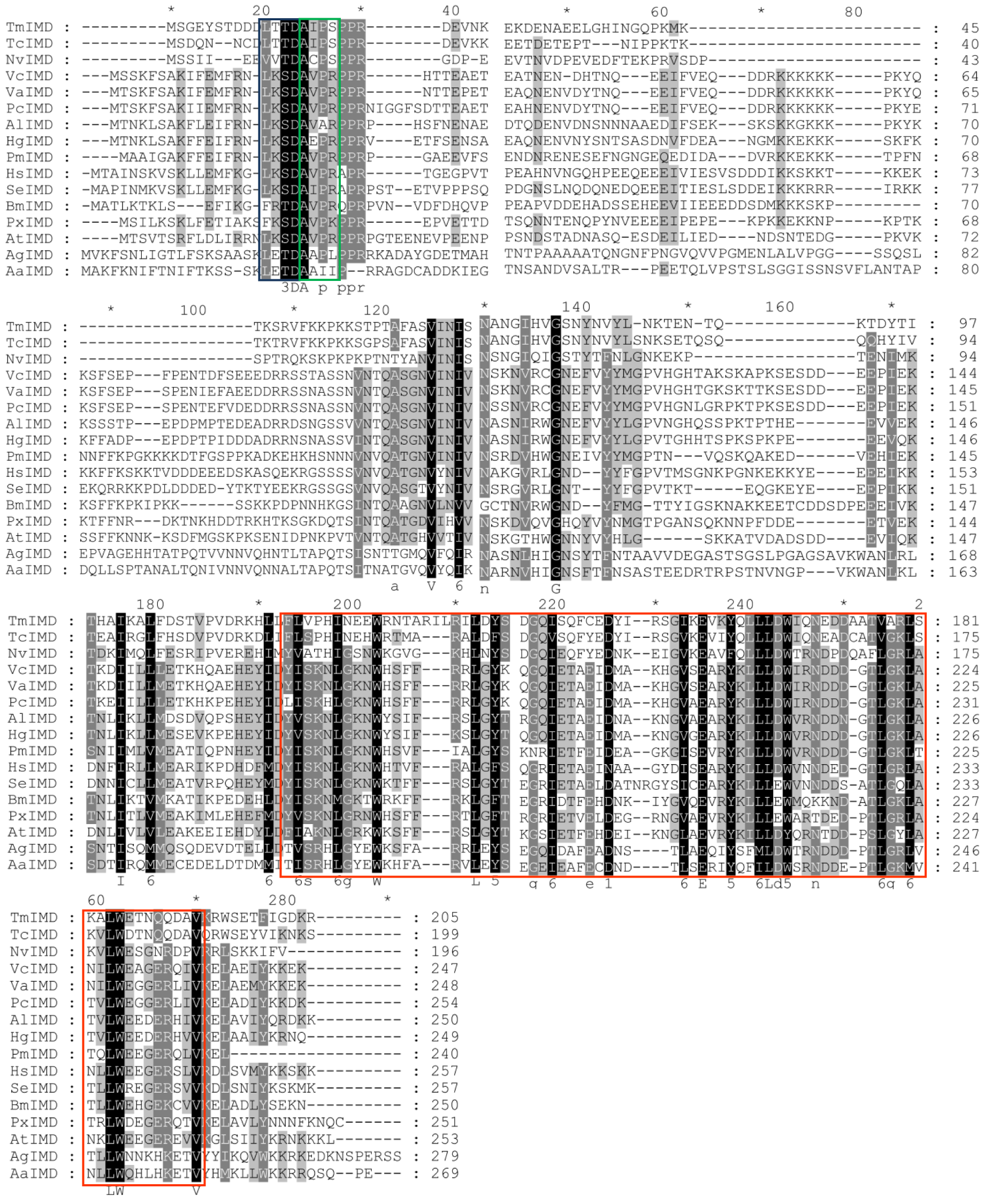


**Figure S1.** An amino acid sequence alignment of 16 IMD proteins from representative insect groups. Shaded sequences represent different levels of conservation, black shading indicates highly conserved residues across species. Green box with solid line: Inhibitor of apoptosis (IAP)-binding motif (IBM); Black box with solid line: caspase cleavage site. The death domain conserved sequences are boxed red. The abbreviations and GenBank accession numbers (in parenthesis) are: TcIMD, *Tribolium castaneum* IMD (XP_008199405); DmIMD-A, *Drosophila melanogaster* IMD, isoform A (NP_573394.1); DmIMD-B, *Drosophila melanogaster* IMD, isoform B (NP_001286572.1); HgIMD, *Hebomoia glaucippe* IMD (AFK75936.1); AgIMD, *Anopheles gambiae* (XP_001688608); PcIMD, *Polygonia c-album* IMD (AFK75940.1); VcIMD, *Vanessa cardui* IMD (AFK75942.1); VaIMD, *Vanessa atalanta* IMD (AFK75941.1); AaIMD, *Aedes aegypti* AAEL010083-PA (EAT37980.1); AlIMD, *Appias lyncida* IMD (AFK75935.1); SgIMD, *Schistocerca gregaria* IMD-like protein (AFK75938.1); HsIMD, *Heliothis subflexa* IMD (AFK75939.1); PxIMD, *Plutella xylostella* IMD (AFK75937.1); AmIMD, *Apis mellifera* IMD (NP_001157189.1); PcIMD, *Polygonia c-album* IMD-like protein (AFK75940.1); and NvIMD, *Nasonia vitripennis* IMD (NP_001135910.1).

**
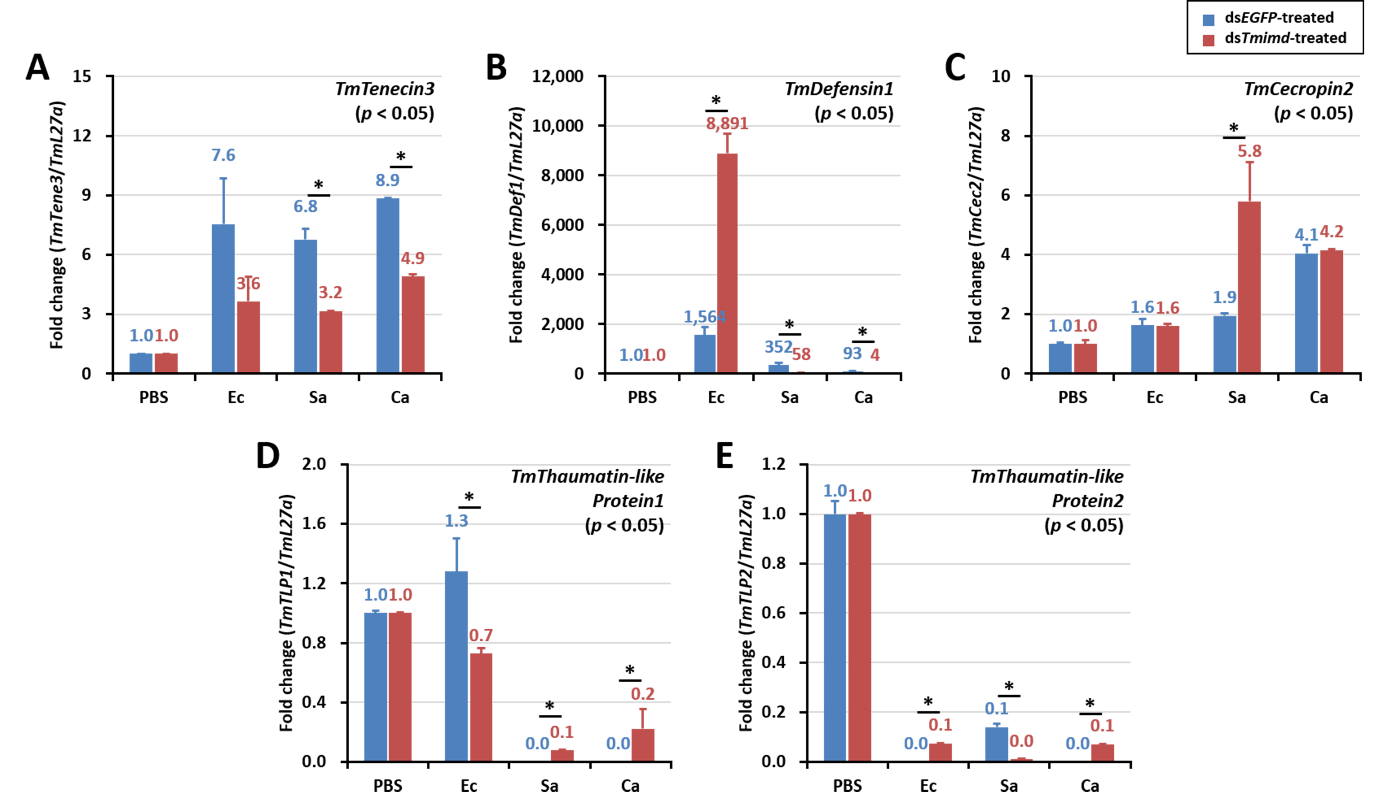
**

**Figure S2.** Impact of *Tmimd* knockdown on AMP expression levels upon microorganism challenge. *E. coli* (Ec), *S. aureus* (Sa), or *C. albicans* (Ca) were injected into ds*Tmimd*-treated *T. molitor* larvae. The mRNA expression levels of *TmTenecin3* (A), *TmDefensin1* (B), *TmCecropin2* (C), *TmThaumatin-like protein1* (D), and *TmThaumatin-like protein2* (E) were measured by qPCR. *EGFP* dsRNA was injected as a negative control and *TmL27a* was used as an internal control. All experiments were performed three times with similar results. All data were analyzed using Student’s *t*-test (* *p* < 0.05).
